# Antiskin Inflammatory Activity of Black Ginger *(Kaempferia parviflora)* through Antioxidative Activity

**DOI:** 10.1155/2018/5967150

**Published:** 2018-04-03

**Authors:** Myung-hee Lee, Ah-Ram Han, Mi Jang, Hyo-Kyoung Choi, Sung-Young Lee, Kyung-Tack Kim, Tae-Gyu Lim

**Affiliations:** ^1^Korea Food Research Institute, Wanju-gun, 55365 Jeollabuk-do, Republic of Korea; ^2^The Hormel Institute, University of Minnesota, 801 16th Ave NE, Austin, MN 55912, USA

## Abstract

*Kaempferia parviflora* (Krachaidum (KD)) is a traditional herbal medicine and has properties that are beneficial for human health. In the current study, we sought to investigate the anti-inflammatory properties of KD extract (KPE). In mouse skin tissue, UV light representing solar wavelengths (sUV) increased COX-2 expression, while treatment with KPE reduced this effect. The anti-inflammatory activity of KPE was confirmed in *in vitro* models. MAPK signaling pathways were activated by sUV irradiation, and this was also repressed in the presence of KPE treatment. It is assumed that the anti-inflammatory activity of KPE is caused by the antioxidative effect. Furthermore, we confirmed the critical role of oxidative stress in sUV-induced COX-2 expression. We analyzed the polyphenol composition of KPE. Of the polyphenols identified, gallic acid, apigenin, and tangeretin were identified as the major polyphenols (at 9.31 ± 1.27, 2.37 ± 0.14, and 2.15 ± 0.19 *μ*g/mg dry weight, resp.). Collectively, these findings show that in the presence of sUV irradiation, KD has anti-inflammatory properties and antioxidative effects in the skin.

## 1. Introduction

*Kaempferia parviflora* (Krachaidum (KD)), commonly known as Thai ginseng, is a member of the Zingiberaceae family [1]. It has long been used as a popular ingredient in health tonics in Thailand, where it is locally known as *Kra-chai-dam*. It has been reported that the phytochemical components of KD include flavones, flavonol glycosides, phenolic glycosides, and *β*-sitosteryl myristate [2]. Various studies have also reported that KD exhibits anti-inflammatory [3], antiallergenic [4], antimycobacterial, antigastric ulcer [5], and antimutagenic activities [6].

Of the various environmental factors that affect the skin, UV light (the major natural source being sunlight) is the most important. Solar UV induces photochemical reactions, which are known to be a major cause of inflammatory skin lesions and skin cancer [7]. The wavelengths within UV are divided into three bands: UVA (320–400 nm), UVB (280–320 nm), and UVC (200–280 nm) [8]. The UV components of sunlight that reach the Earth's surface (daylight UV) are UVA and UVB (290–400 nm). It has been reported that after UV light irradiation, high concentrations of reactive oxygen species can be generated, which then induces cell membrane damage and skin inflammation [9]. As skin cancer has emerged as a major subject of research efforts, numerous studies on the protection of skin from the harmful effects of sunlight have been conducted [9–11].

PGE_2_ is a widely known prostaglandin (PG) that is known to play an important role as a mediator of acute inflammation and as a regulator of immune response [12, 13]. Prostaglandin synthesis is promoted by the transcription factor cyclooxygenase (COX). COX exists as two isoenzymes including COX-1, which is continuously expressed and is involved in regulating homeostasis-related functions in the body [14], and COX-2, which is expressed in the presence of stimulatory conditions such as inflammation. In addition, the prostaglandin generated by COX-2 is known to be involved in the inflammatory response as well as cell proliferation [15].

In the current study, we obtained an extract of KD and investigated its anti-inflammatory effects using *in vitro* and *in vivo* models.

## 2. Materials and Methods

### 2.1. Chemicals and Reagents

MTS solution was obtained from Promega (Madison, WI, USA). Dulbecco's modified eagle medium (DMEM), fetal bovine serum (FBS), and penicillin/streptomycin were purchased from Thermo Fisher Scientific (San José, CA, USA). Primary antibodies specific for COX-2, phosphorylated MKK4 (Ser^257^/Thr^261^), phosphorylated SAPK/JNK (Thr^183^/Tyr^185^), phosphorylated MKK3 (Ser^189^)/6 (Ser^207^), phosphorylated p44/p42 ERK1/2 (Thr^202^/Tyr^204^), total MKK3, phosphorylated c-Jun (Ser^73^), phosphorylated MEK (Ser^217/221^), phosphorylated p90RSK (Thr^573^), phosphorylated p38 (Tyr^180/182^), and total p38 were purchased from Cell Signaling Technology (Danvers, MA). Antibodies against total ERK1/2, total MKK4, total JNK, and total MEK were obtained from Santa Cruz Biotechnology (Santa Cruz, CA, USA).

### 2.2. Extract Condition

Approximately 1 g of KD powder sample was obtained from Thanyaporn (Samutprakarn, Thailand). The powder was mixed with 25 mL of 80% (*v*/*v*) EtOH. The soluble components were then extracted in 80°C hot water using a reflux condenser. The extract was filtered through filter paper number 2 (Whatman, Maidstone, England) and vacuum-concentrated and subsequently dissolved in 5 mL of distilled water and freeze-dried, for use as samples for the analysis of antioxidant test.

### 2.3. UPLC-MS/MS Analysis

The analyses were performed using an ACQUITY UPLC system (Waters, Miliford, MA, USA) with ACQUITY UPLC BEH C18 columns (2.1 mm × 100 mm, 1.7 *μ*m). The mass spectrometer was a Waters Xevo TQ triple-quadrupole mass spectrometer equipped with electrospray ionization (ESI) mode. MassLynx 4.1 (Waters) software was used for data processing. The mobile phase included 0.1% formic acid aqueous solution (solvent A) and 0.1% formic acid in acetonitrile (solvent B), and a gradient elution program was performed: 0–10 min, 99–70% solvent A; 10–12 min, 70–5% solvent A; 12–14 min, 5–99% solvent A; and 14–20 min, 99% solvent A. The flow rate was set at 0.65 mL/min, and the column temperature was kept at 40°C, with a total run time of 20 min. The autosampler was conditioned at 4°C, and the injection volume was 5 *μ*L. The LC-MS/MS system was operated in negative ESI mode and scanned using multiple reaction monitoring (MRM) mode. The voltages for capillary, cone, and collision energy were set at 2.5 kV, 20 V, and 24 V, respectively. The gas flow for desolvation and cone was 800 and 50 L/h, respectively. The source temperature and desolvation gas temperature were 150 and 400°C, respectively.

### 2.4. Cell Culture

JB6 P^+^ and HaCaT cells were cultured in DMEM supplemented with 10% FBS and 0.1% penicillin/streptomycin/neomycin at 37°C in a humidified atmosphere of 5% CO_2_. Stable transfectants were generously provided from the Dr. Zigang Dong laboratory in Hormel Institute. Luciferase reporter-transfected JB6 P^+^ cells were constructed as described previously [16].

### 2.5. Solar UV Irradiation Systems

UVA-340 lamps were purchased from Q-Lab Corporation (Cleveland, OH). The UVA-340 lamps provide the best possible simulation of sunlight in the critical short-wavelength region from 365 nm down to the solar cutoff of 295 nm with a peak emission of 340 nm. The percentage of UVA and UVB emitted by the UVA-340 lamps was measured by a UV meter at 94.5% and 5.5%, respectively.

### 2.6. Cell Viability

The cytotoxicity of the sample was measured using CellTiter96 Aqueous One Solution (Promega, Madison, WI). The cells were cultured to confluence in 96-well plates. KPE was then treated to the cells for 24 h. 20 *μ*L of MTS solution was then added for 1 h at 37°C in a 5% CO_2_ incubator. The absorbance was evaluated at 492 nm.

### 2.7. Luciferase Assay for the COX-2 Promoter and NF-*κ*B Transcriptional Activity

JB6 P^+^ cells stably transfected with COX-2, and NF-*κ*B luciferase reporter plasmids were used for the luciferase assay. Cells reached confluence before the serum was replaced with serum-free DMEM for 24 h. KPE was pretreated to the cells for 1 h. Then, 23 kJ/cm^2^ of sUV was irradiated to the cells and the cells were incubated at 37°C in a humidified atmosphere of 5% CO_2_. After 4 h of sUV exposure, the cells were disrupted using lysis buffer (0.1 M potassium phosphate buffer (pH 7.8), 1% Triton X-100, 1 mM DTT, and 2 mM EDTA), and luciferase activity was measured using a luminometer (Luminoskan Ascent, Thermo Electron, Helsinki, Finland).

### 2.8. Western Blot

JB6 P^+^ and HaCaT cells were cultured to 100% confluency, and the serum was removed for 24 h to eliminate FBS-mediated signaling activation. KPE was then treated to the cells at various concentrations. After 1 h of incubation with KPE, the cells were exposed to sUV (23 kJ/cm^2^), before lysis using 1x lysis buffer (Cell Signaling Biotechnology, Beverly, MA). Equal concentrations of the protein samples were separated on polyacrylamide gels (Bio-Rad Laboratories, Hercules, CA) and transferred to Immobilon-P membranes (Millipore, Billerica, MA). The membranes were blocked with 5% fat-free milk for 1 h and incubated with specific primary antibodies at 4°C overnight. The proteins were then hybridized with HRP-conjugated secondary antibodies and detected using a chemiluminescence detection kit (GE Healthcare, Pittsburgh, PA).

### 2.9. PGE2 Assay

KPE was treated to JB6 P^+^ cells for 1 h, before sUV (23 kJ/cm^2^) was irradiated to the cells. After 4 h, the production of PGE2 was measured from the cell culture media using a PGE2 enzyme immunoassay kit (R&D Systems, Minneapolis, MN, USA). The experiments were performed in triplicate.

### 2.10. ABTS and DPPH Radical Scavenging Assay

The ABTS radical-scavenging activity of KPE was measured using a slightly modified method from van den Berg et al. [17]. 0.1 M PBS (pH 7.4), 2.5 mM ABTS [2,2′-azino-bis-(3-ethylbenzothiazoline-6-sulfonic acid)], and 1.0 mM AAPH [2,2′-azobis(2-mehtylpropionamidine) dihydrochloride] were mixed and maintained for 12 min in a dark room at 68°C, then quickly cooled to generate ABTS radical solution. For assessment of the antioxidant capacity, 20 *μ*L of the KD extract was mixed with 980 *μ*L of ABTS radical solution and incubated for 10 min at 37°C, whereupon absorbance was measured using multimode microplate readers (Infinite® 2000 PRO, Tecan, Switzerland) at 734 nm. DPPH radical scavenging activity was measured as follows; each 0.2 mL of the KD extract was added to 3 mL of ethanol, to which 0.8 mL of 4 × 10^−4^ M DPPH dissolved in ethanol was added. This mixture was vortexed for 10 s and maintained at room temperature for 10 min, and the absorbance was measured at 517 nm [18]. The DPPH radical scavenging activity was expressed as a percentage of the absorbance of the group to which no DPPH was added. All experiments were performed at least in triplicate.

### 2.11. ORAC Assay

The ORAC assay was performed by KOMABIOTECH (Seoul, Korea). The total procedure was followed by the manufacturer's instruction. Briefly, the oxygen radical absorbance capacity was measured by the fluorescence unit. The ORAC activity was represented using Trolox equivalent antioxidant capacity. The experiments were performed in triplicate.

### 2.12. Animal Study

The animal experimental protocol (2017-0030) was approved, and animals were maintained under specific pathogen-free conditions based on the guidelines established by the Experimental Animal Research Laboratory of the Korea Food Research Institute. Female SKH-1 hairless mice (5 weeks old, 5 mice in each group) were obtained from Central Lab, Animal Inc. (Seoul, Korea). The animals were housed in climate-controlled quarters (24°C at 50% humidity) with a 12 h light/dark cycle.

For the experiment, 50 and 100 mg/kg b.w. of KPE in DMSO were applied to the dorsal skin of mice for 1 h, before the animals were exposed to 46 kJ/cm^2^ of sUV. The mice were sacrificed after 4 h, and the dorsal skin sections were collected and the protein was recovered using RIPA buffer.

### 2.13. Immunohistochemical Staining

Immunohistochemical staining was performed by Abion CRO (Seoul, Korea). Briefly, the skin tissue samples were fixed in 10% formalin before dehydration using a graded ethanol series. In accordance with general protocols, the tissue was processed for embedding in paraffin wax. To exclude endogenous peroxidase activity, the sections were incubated in 0.3% H_2_O_2_ for 15 min, and then each primary antibody was treated at a 1 : 200 dilution factor for 1 h. The detection system visualizing anti-mouse antibodies (K4001; DAKO, Glostrup, Denmark) was used according to the manufacturers' instructions. Slides were stained with liquid diaminobenzidine tetrahydrochloride (DAB^+^), a high-sensitivity substrate-chromogen system (K3468; DAKO). The images on the slides were visualized with an Olympus BX40 light microscope.

### 2.14. Statistical Analysis

All experiments were performed at least three times. Data are expressed as the mean and SD values, and Student's *t*-test was used for single statistical comparisons. A probability cutoff of *p* < 0.05 was used as the criterion for statistical significance.

## 3. Results

### 3.1. *Kaempferia parviflora* Extract Counteracts UV-Induced Inflammatory Activity in the Skin of Hairless Mice

**Figure 1:**
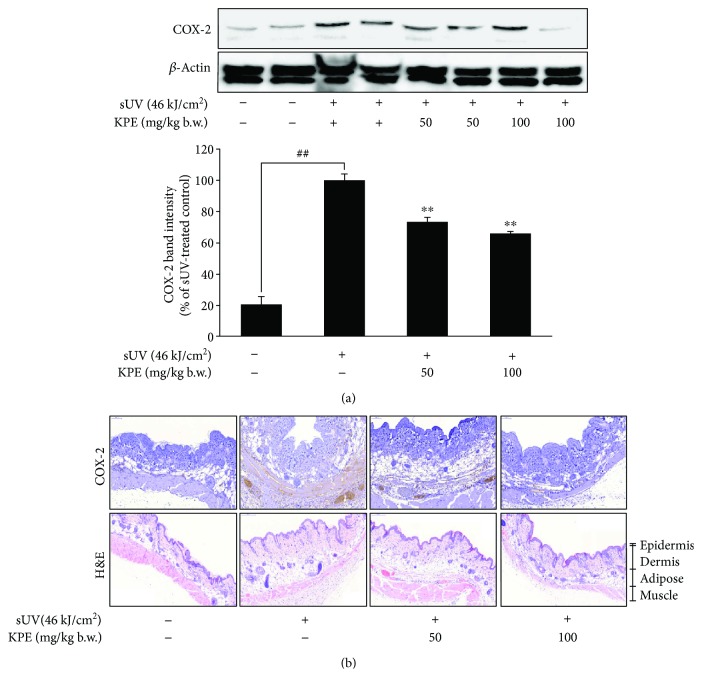
Inhibitory effect of *Kaempferia parviflora* extract (KPE) on sUV-induced COX-2 expression in mouse skin. (a) Each protein level was detected with the specific primary antibodies. The detailed procedure was described in Materials and Methods. Relative protein expression levels are expressed as the percentage of sUV-treated group intensity, which was set to 100%. The pound signs (##) and asterisks (∗∗) indicate significant difference (of *p* < 0.001) compared to the untreated group and sUV-treated group, respectively. (b) Histopathological analysis of hematoxylin and eosin (H&E) and COX-2 expression in mouse skin tissue. The detailed procedure is described in Materials and Methods.

COX-2 is a representative inflammatory biomarker [15]. Several studies have reported that aberrant increments in COX-2 expression are associated with tumor promotion. Thus, we examined the effect of KPE on sUV-induced COX-2 expression using a hairless mouse model. Briefly, KPE (50 and 100 mg/kg b.w.) was applied to the dorsal skin of mice for 1 h, before the animals were exposed to 46 kJ/cm^2^ of sUV. As seen in Figure 1(a), KPE reduced sUV-induced COX-2 expression and the suppression was confirmed in immunohistological staining (Figure 1(b)).

### 3.2. *Kaempferia parviflora* Extract Inhibits Solar UV-Induced PGE2 Production via a Reduction in COX-2 Expression in Mouse Epidermal Cells

**Figure 2:**
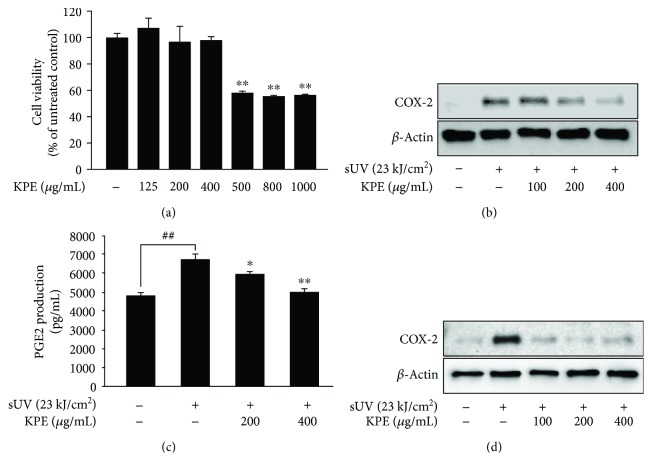
Inhibitory effect of *Kaempferia parviflora* extract (KPE) on sUV-induced prostaglandin E2 (PGE2) production via COX-2 reduction in JB6 P^+^ and HaCaT cells. (a) The cytotoxicity of KPE was measured using MTS analysis as indicated in Materials and Methods. The COX-2 expression and PGE2 production were determined with Western blot analysis and PGE2 assay in JB6 P^+^ (b, c) and HaCaT (d). KPE was pretreated to the cells for 1 h before sUV (23 kJ/cm^2^) exposure. After 3 h, the protein was collected. The pound signs (##) and asterisks (∗∗) indicate significant difference (*p* < 0.001) compared to the untreated group and sUV-treated group, respectively. And the asterisk (∗) represents a significant difference (*p* < 0.01) compared to the sUV-treated group.

Production of PGE2 is closely associated with the release of cytokines, such as interleukin- (IL-) 1*α* and tumor necrosis factor- (TNF-) *α* [19]. We evaluated the effect of KPE on sUV-induced PGE2 production in JB6 P^+^ cells. The mouse epidermal JB6 P^+^ cell line is a well-established cell line for assessing novel anti-inflammatory agents. Similar with findings from a previous study [19], sUV irradiation statistically enhanced PGE2 production in JB6 P^+^ cells, while pretreatment with KPE attenuated sUV-induced PGE2 production in a dose-dependent manner (Figure 2(c)). The dose range of KPE did not exhibit any cell cytotoxicity up to concentrations of 400 *μ*g/mL (Figure 2(a)). Additionally, sUV-induced COX-2 expression was downregulated by KPE treatment (Figure 2(b)). To obtain more physiologically relevant findings, we assessed the effect of KPE on human keratinocyte HaCaT cells. The inhibitory effect of KPE on sUV-induced COX-2 expression was also reflected in the HaCaT cells (Figure 2(d)).

### 3.3. *Kaempferia parviflora* Extract Modulates sUV-Induced COX-2 Expression via Transcriptional Regulation

**Figure 3:**
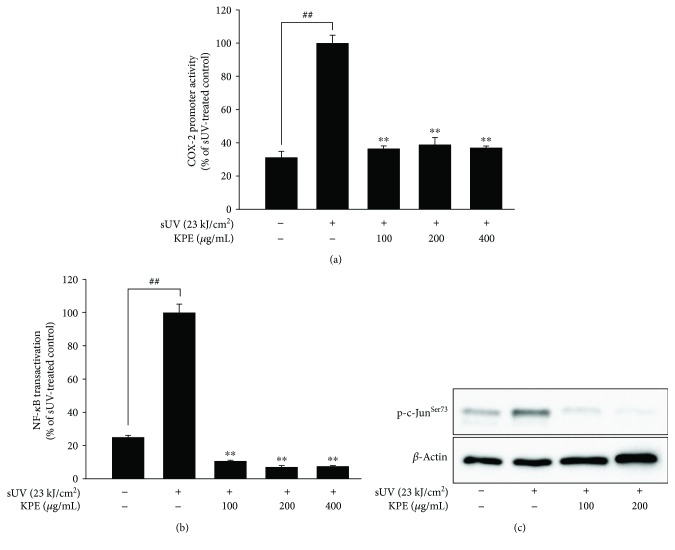
Transcriptional regulation of *Kaempferia parviflora* extract (KPE) on sUV-induced COX-2 expression. The promoter and transcriptional activity of *cox-2* (a) and NF-*κ*B (b) were downregulated by KPE, respectively. The detailed procedure is indicated in Materials and Methods. Briefly, KPE was pretreated to the cells prior to sUV (23 kJ/cm^2^) irradiation. After 3 h of sUV exposure, each luciferase activity was measured. The pound signs (##) and asterisks (∗∗) indicate significant difference (of *p* < 0.001) compared to the untreated group and sUV-treated group, respectively. (c) The phosphorylation level of c-Jun was detected using Western blot assay with a specific primary antibody. Data are representative of 3 independent experiments that gave similar results.

It has been reported that mutated DNA-binding sites within NF-*κ*B and AP-1 can cause a significant attenuation of *cox-2* gene expression [20]. AP-1 and NF-*κ*B are major transcription factors for sUV-induced COX-2 expression [21]. We evaluated the transcriptional activity of NF-*κ*B as well as *cox-2* promoter activity using stably transfected JB6 P^+^ cells. In Figure 3(a), the promoter activity of *cox-2* was enhanced after 3 h of sUV exposure, and treatment with KPE for 1 h effectively reduced the *cox-2* promoter activity down to control levels.  Figure 3(b) shows a similar trend seen in the *cox-2* promoter assay. The irradiation with sUV increased NF-*κ*B transcriptional activity, while KPE treatment suppressed this increment (Figure 3(b)). AP-1 is a complex comprised of Jun family (c-Jun, JunB, and JunD) and Fos family members (c-fos, FosB, Fra-1, and Fra-2) and is generally present as a heterodimer of c-Jun and c-Fos [22]. Thus, we confirmed whether KPE had any effect on c-Jun phosphorylation. As shown in Figure 3(c), the phosphorylation of c-Jun at Ser^73^ was elevated by sUV, and this increment was suppressed by KPE treatment.

### 3.4. *Kaempferia parviflora* Extract Inhibits sUV-Induced MKK4-JNK, MKK3/6-p38, and the MEK-ERK Signaling Pathway

**Figure 4:**
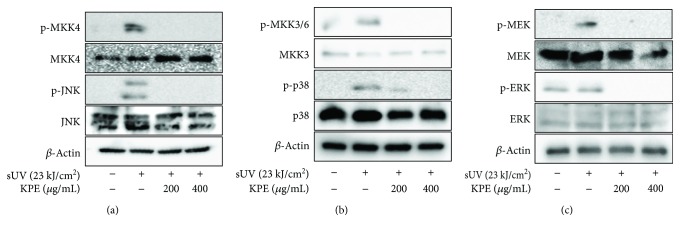
Inhibitory effect of *Kaempferia parviflora* extract (KPE) on sUV-induced MKK4-JNK (a), MKK3/6-p38 (b), and MEK-ERK (c) signaling pathway in JB6 P^+^ cells. The detailed method is presented in Materials and Methods. Briefly, KPE was pretreated to the cells for 1 h, and the sUV irradiation step was followed. Each protein level was estimated with primary antibodies. Data are representative of 3 independent experiments that gave similar results.

**Figure 5:**
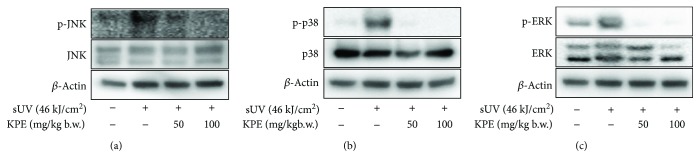
Effect of *Kaempferia parviflora* extract (KPE) on the sUV-induced MAPK signaling pathway in mouse skin. 50 and 100 mg/kg b.w. of KPE were treated to the abdominal skin of female SKH-1 hairless mice for 1 h. Subsequently, 46 kJ/cm^2^ of sUV was exposed to the mice. The protein level was quantified using Western blot analysis. Data are representative of 3 independent experiments that gave similar results.

The mitogen-activated protein kinase (MAPKs) family is a representative inflammatory signaling pathway that regulates sUV-induced COX-2 expression [8, 23]. MAPK kinases (MAP2Ks), MKK4, MKK3/6, and MEK directly activate MAPKs, JNK, p38, and ERK, respectively [24]. Thus, we confirmed the effect of KPE on sUV-induced MAPK activation. As seen in Figure 4, irradiation of sUV activated the MAPKK-MAPK signaling pathway, and pretreatment with KPE significantly alleviated MKK4-JNK, MKK3/6-p38, and MEK/ERK phosphorylation (Figure 4) in JB6 P^+^ cells. To confirm these findings *in vivo*, 50 and 100 mg/kg b.w. treatments of KPE were applied to the dorsal skin of mice prior to sUV irradiation. It was observed that the phosphorylation of all MAPKs was downregulated in the presence of KPE treatment (Figure 5).

### 3.5. *Kaempferia parviflora* Extract Exhibits Antioxidative Activity

**Figure 6:**
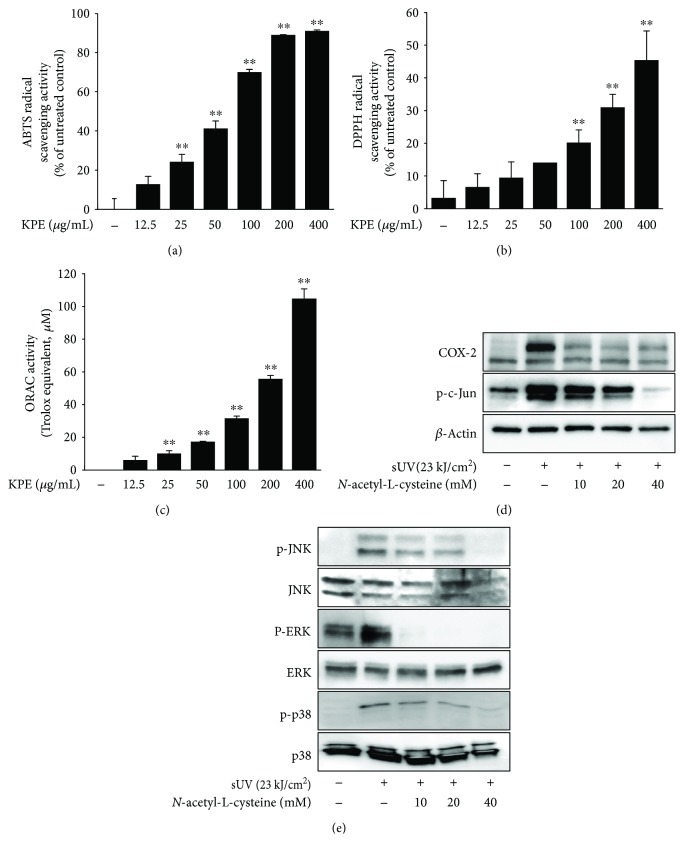
Antioxidative effect of *Kaempferia parviflora* extract (KPE) and the involvement of oxidative stress in sUV-induced inflammatory mechanisms. The antioxidative effect of KPE was measured using ABTS (a), DPPH (b), and ORAC (c) assay. (d) *N*-acetyl-L-cysteine (NAC) suppressed sUV-induced COX-2 expression (d) and MAPK phosphorylation (e). NAC was treated to the cells prior to 1 h of sUV irradiation. Each protein amount was measured using Western blot analysis with specific primary antibodies. Data are representative of 3 independent experiments that gave similar results. The asterisks (∗∗) indicate the significant difference (of *p* < 0.001) compared to the untreated control.

Excessive production of free radicals is harmful to the human body and enhances inflammatory signaling pathways [25, 26]. Thus, suppressing agents against aberrant ROS production have been developed to attenuate inflammation [27–29]. Because KPE inhibited sUV-induced inflammatory signaling pathways, we sought to confirm the antioxidative activity using ABTS radicals. As seen in Figures 6(a) and 6(b), KPE exhibited dose-dependent ABTS and DPPH radical scavenging activity. Of particular note, almost 90% of the ABTS radicals were destroyed by 200 *μ*g/mL of KPE treatment (Figure 6(a)). ORAC assay was also performed to verify the antioxidative activity of KPE. As seen in Figure 6(c), the dose-dependent Trolox equivalent antioxidant capacity was represented by KPE treatment. To evaluate the role of oxidative stress in sUV-induced inflammation, we used *N*-acetyl-L-cysteine (NAC), a well-known antioxidant chemical. Interestingly, we found a dramatic reduction of sUV-induced COX-2 expression by NAC treatment. Furthermore, the sUV-activated MAPK signaling pathway was significantly blocked by NAC treatment. Overall, we assumed that sUV exposure increases the inflammatory status by oxidative stress and KPE attenuated sUV-related inflammatory condition through antioxidative activity. Next, we analyzed the polyphenolic composition of KPE using UPLC-MS/MS analysis (Supplementary Figure 1). Interestingly, KPE contained gallic acid (9.31 ± 1.27 *μ*g/mg dry weight), apigenin (2.37 ± 0.14 *μ*g/mg dry weight), and tangeretin (2.15 ± 0.19 *μ*g/mg dry weight) (Table 1).

## 4. Discussion

In the present study, an extract of *Kaempferia parviflora* (KPE) exhibited an attenuating effect on sUV-induced COX-2 expression in murine dorsal skin sections (Figure 1). In addition, inflammatory MAPK signaling pathways were blocked in the presence of pretreatment with KPE. In 2009, Sae-wong et al. reported that KPE elicits anti-inflammatory activity via the suppression of iNOS and COX-2 mRNA expression [3]. We further confirmed this suppressive effect on COX-2 *in vitro* (Figure 2). KPE markedly inhibited sUV-induced COX-2 expression in keratinocytes sourced from human skin, suggesting that KPE may be useful in suppressing sunlight-mediated inflammation in human skin. The underlying mechanisms of the effect of KPE were shown to involve transcriptional regulation of COX-2 (Figure 3). Similar with our *in vivo* findings (Figure 5), the phosphorylation of various MAPKs was downregulated by KPE, as well as upstream regulators of the MAPKs. Notably, KPE demonstrated a potent dose-dependent radical scavenging effect (as seen in Figure 6(a)). UV stimulates reactive oxygen (ROS) generation and upregulates the expression of COX-2 and PGE2, which significantly contributes to skin damage and inflammation. In addition, UV-induced ROS activates MAPKs and inflammatory mediator enzymes as well as NF-*κ*B and AP-1 [30, 31]. Because aberrant ROS induction following sunlight exposure is a primary factor in sUV-related skin inflammation, the antioxidative properties of KPE in a sUV-induced skin inflammation model were confirmed. Treatment with NAC, an ROS scavenger, inhibited the sUV-induced COX-2 expression as well as phosphorylation of c-Jun and MAPKs (Figures 6(d) and 6(e)). These results suggest that NAC and KPE have a similar effect by sUV-induced inflammatory responses, and it is associated with antioxidative activity. Collectively, we speculate that KPE may exert anti-inflammatory effects by regulating the intracellular level of oxidative stress.

To identify the bioactive compounds present in KPE, we analyzed its polyphenolic composition using UPLC-MS/MS. The primary compounds identified were gallic acid, apigenin, and tangeretin. The anti-inflammatory activities of gallic acid [32], apigenin [33, 34], and tangeretin [9] have previously been reported. Of particular note, Src kinase has been identified as a direct target of apigenin in a UVB-induced skin inflammation model [34]. Additionally, we have previously confirmed the anti-inflammatory effects of tangeretin in JB6 P^+^ cells in 2011 [9]. Previously, several researches have tried to identify the bioactive compounds in KPE [35, 36]. Horigome et al. reported that 5,6-dimethoxyflavone (DMF) and 5-hydroxy-3,7,3′,4′-tetrymethoxyflavone (TMF) were found in KPE and these two compounds revealed that inhibition of the degranulation and the production and mRNA expression of inflammatory mediators [36].

Although further investigation is needed to more clearly understand the anti-inflammatory effects of KPE, our findings highlight the potential for KPE to be developed as an anti-inflammatory agent with various beneficial effects for skin health.

## Figures and Tables

**Table 1:** Polyphenolic composition of KPE (unit: *μ*g/mg dry weight).

Analytes	Contents
Ferulic acid	1.80 ± 0.40
Isorhamnetin	0.61 ± 0.13
Naringenin	1.55 ± 0.56
Luteolin	1.00 ± 0.23
Protocatechuic acid	0.05 ± 0.02
Caffeic acid	0.86 ± 0.15
Gentisic acid	1.94 ± 0.26
Hydroxybenzoic acid	0.89 ± 0.06
Gallic acid	9.31 ± 1.27
Apigenin	2.37 ± 0.14
Tectorigenin	0.01 ± 0.00
Malic acid	0.57 ± 0.05
Tangeretin	2.15 ± 0.19

All data are presented as mean ± standard deviation (*n* = 3).

## Supplementary Materials

Supplementary Materials The chemical composition of KPE. The analyses were performed using UPLC and mass spectrometry. The detailed procedure is presented in Materials and Methods.
