# Supplementary figures and images for: Antiskin Inflammatory Activity of Black Ginger (Kaempferia parviflora) through Antioxidative Activity
Source: Oxid Med Cell Longev. 2018 Apr 3;2018:5967150. doi: 10.1155/2018/5967150 (PMC5903305; doi:10.1155/2018/5967150)

# SUPPLEMENTARY FIGURE 1

## Standard 1

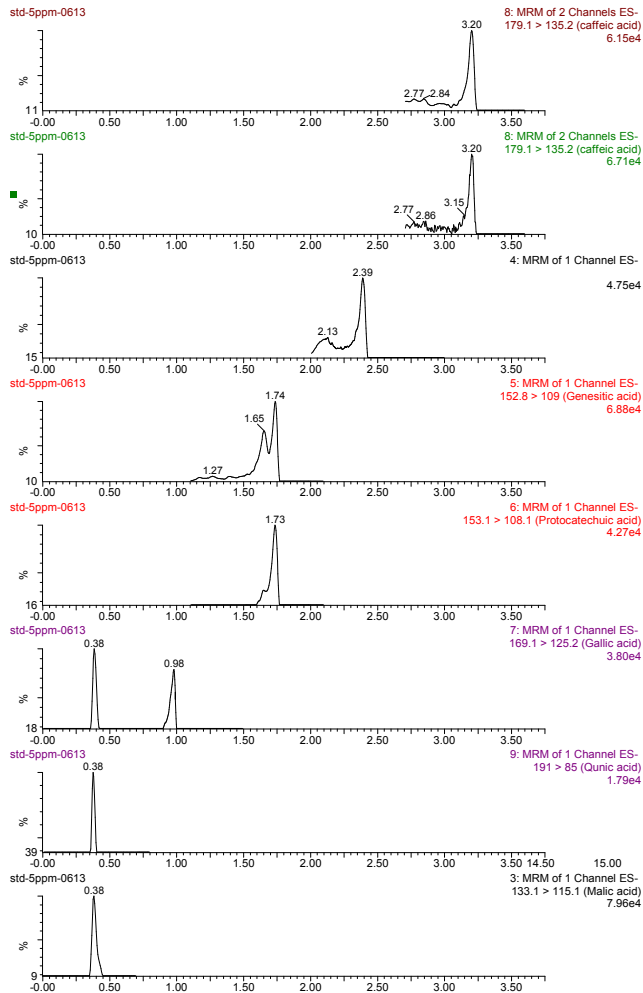

## Standard 2

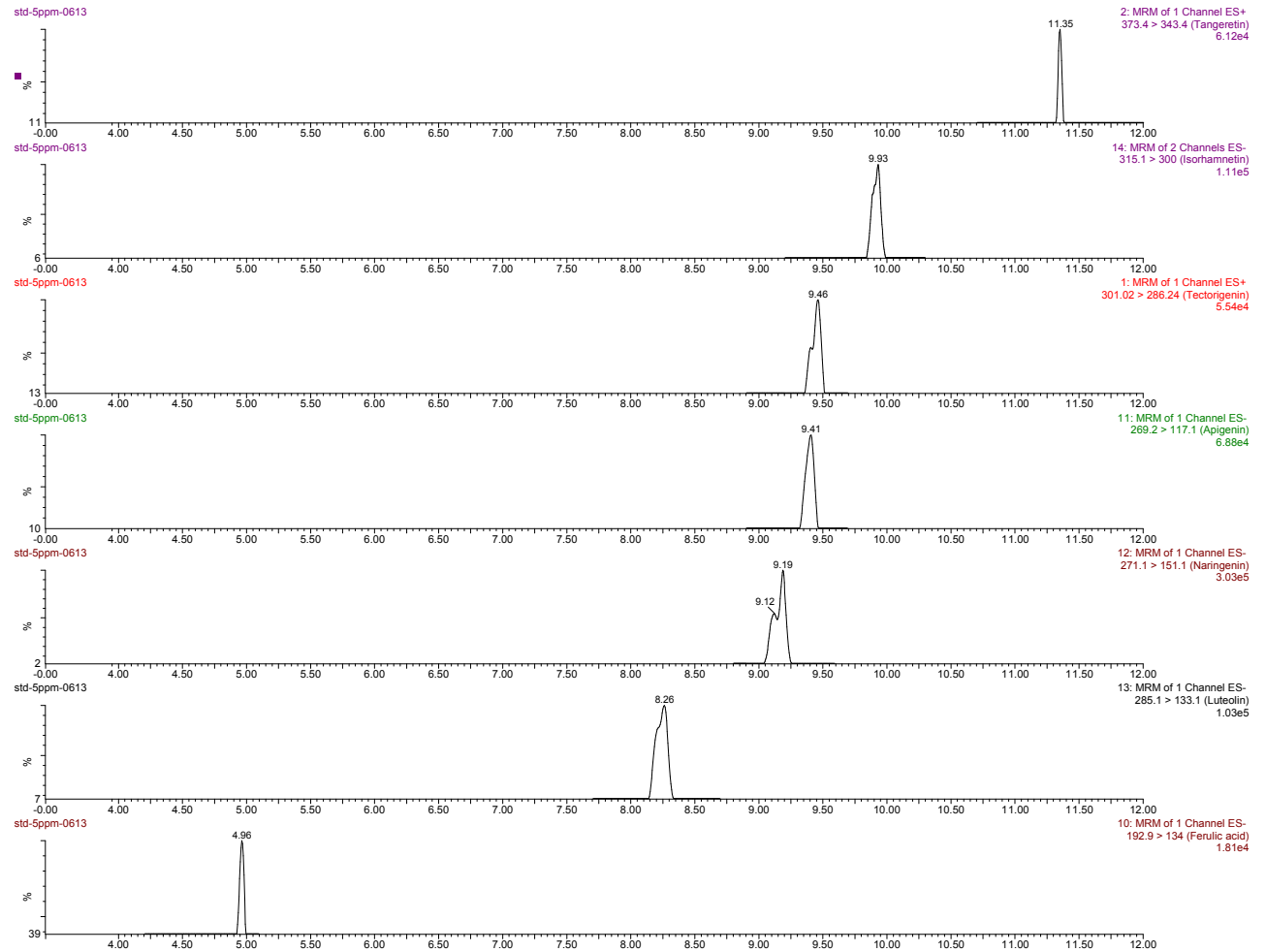

# SUPPLEMENTARY FIGURE 1

## KPE 1

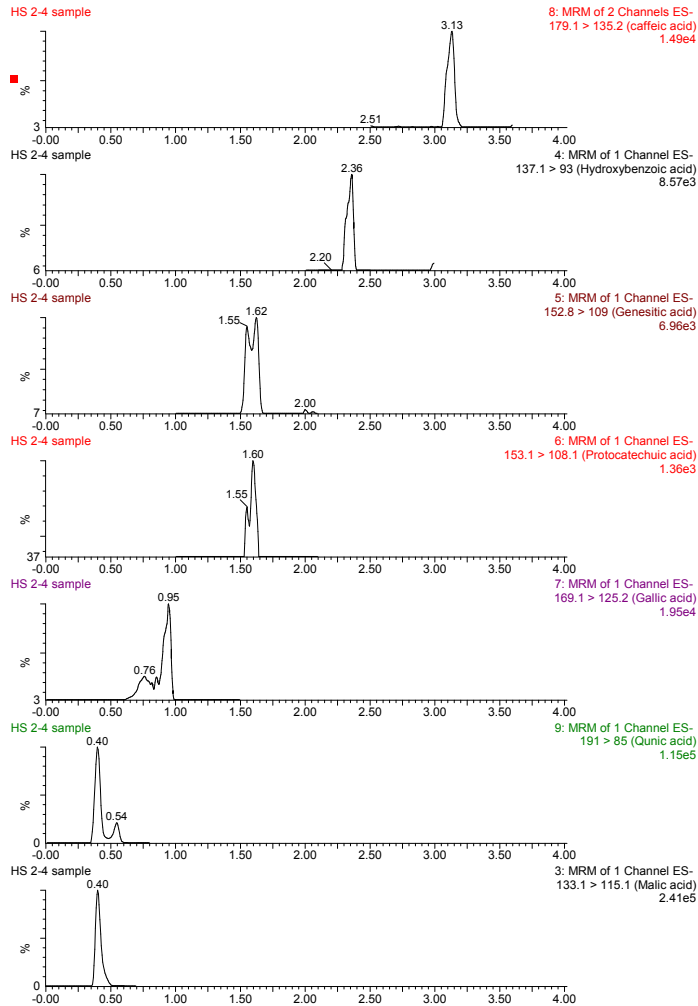

## KPE 2

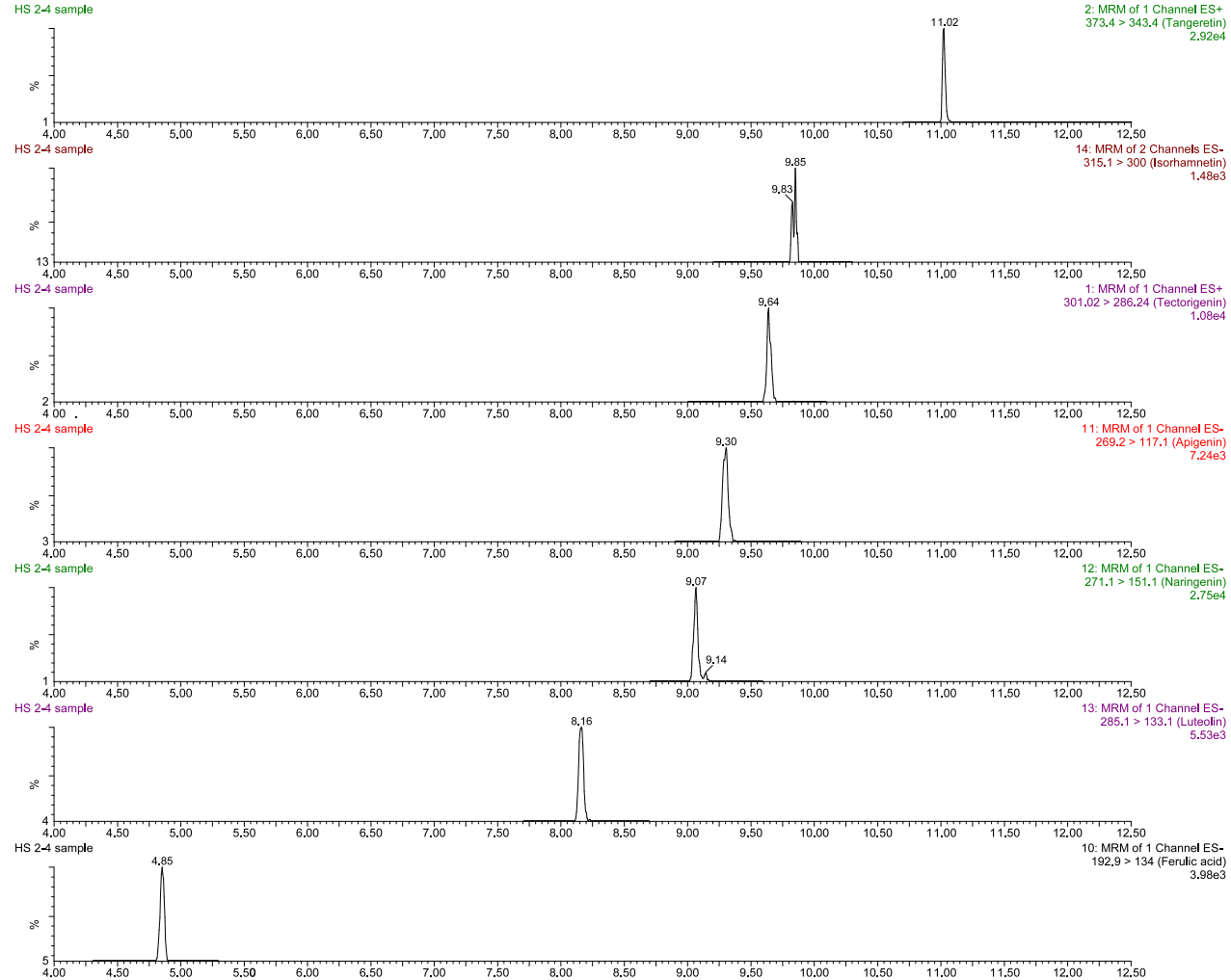

Supplement: Supplementary Materials — The chemical composition of KPE. The analyses were performed using UPLC and mass spectrometry. The detailed procedure is presented in Materials and Methods. [file 5967150.f1.pdf]
